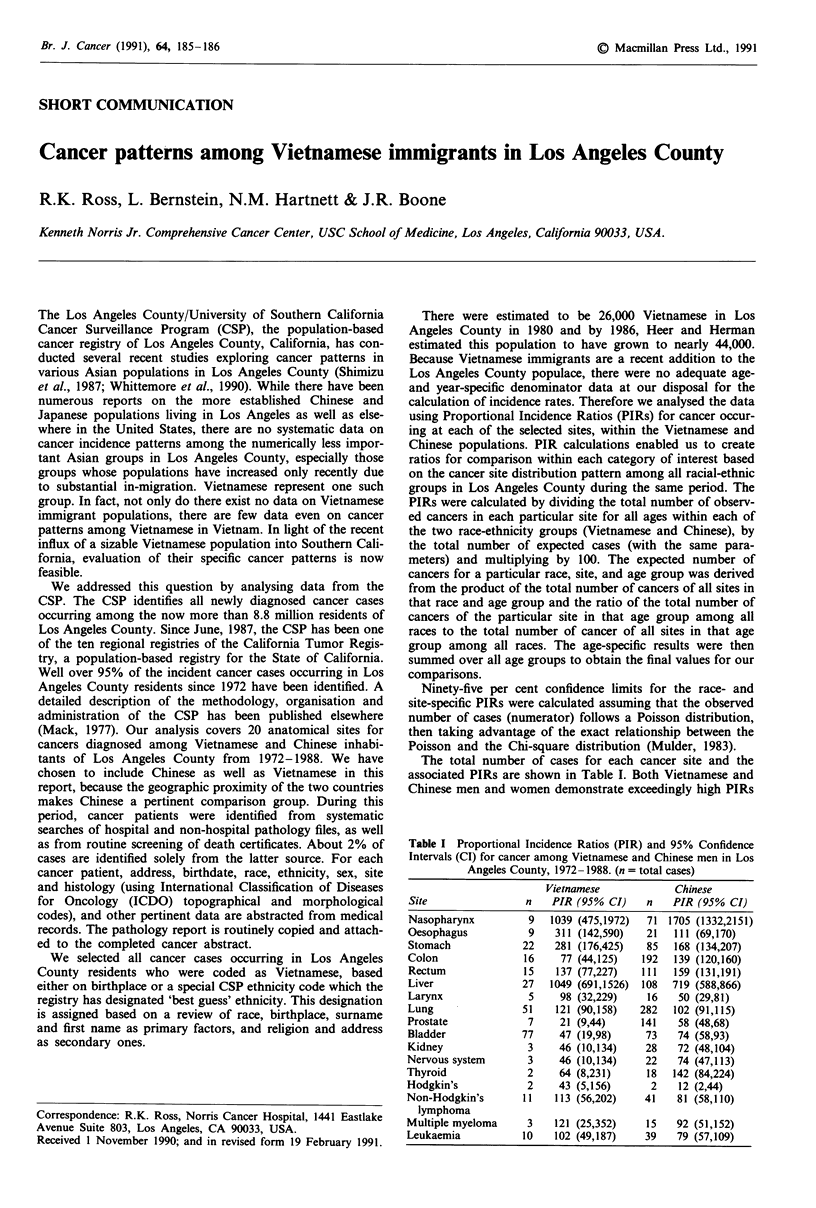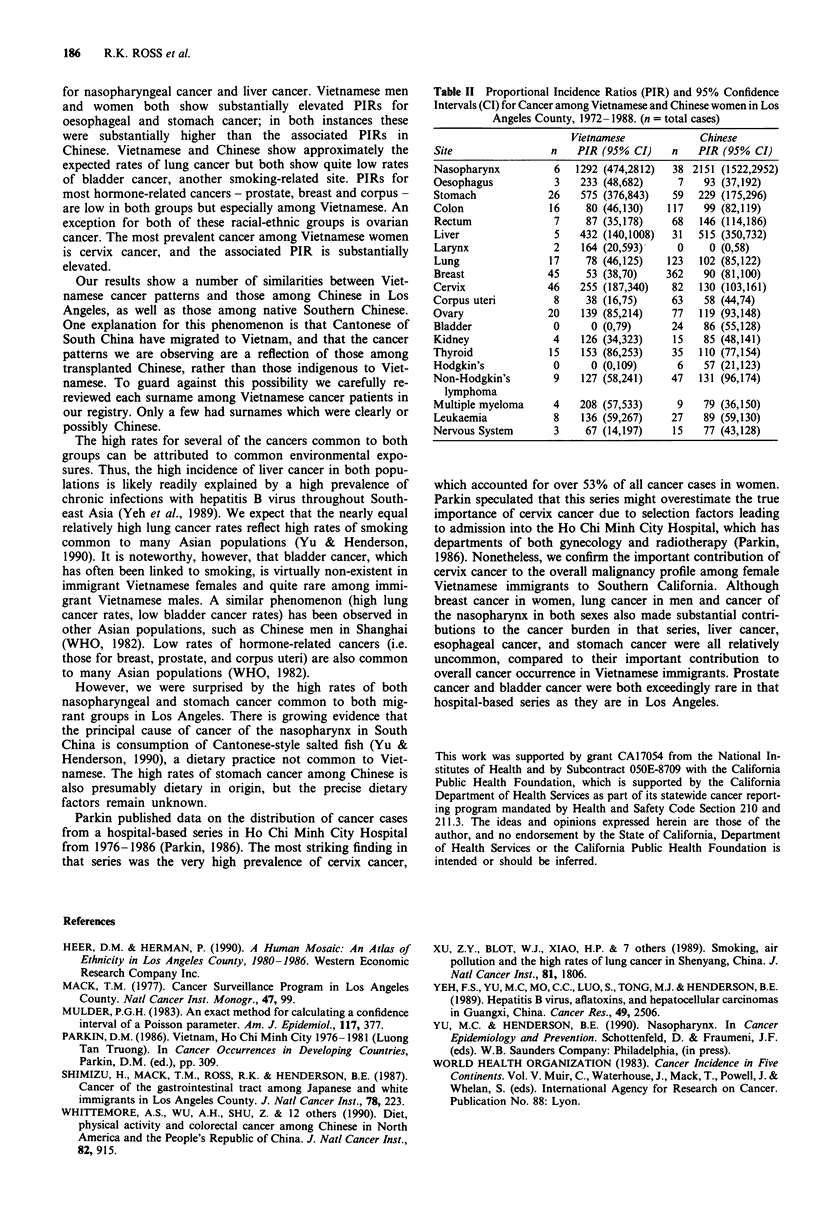# Cancer patterns among Vietnamese immigrants in Los Angeles County.

**DOI:** 10.1038/bjc.1991.267

**Published:** 1991-07

**Authors:** R. K. Ross, L. Bernstein, N. M. Hartnett, J. R. Boone

**Affiliations:** Kenneth Norris Jr. Comprehensive Cancer Center, USC School of Medicine, Los Angeles 90033.


Br. J. Cancer (1991), 64, 185-186 © Macmillan Press Ltd., 1991

SHORT COMMUNICATION

### Cancer patterns among Vietnamese immigrants in Los Angeles County

R.K. Ross, L. Bernstein, N.M. Hartnett & J.R. Boone

*Kenneth Norris Jr. Comprehensive Cancer Center, USC School of Medicine, Los Angeles, California 90033, USA.*

The Los Angeles County/University of Southern California Cancer Surveillance Program (CSP), the population-based cancer registry of Los Angeles County, California, has conducted several recent studies exploring cancer patterns in various Asian populations in Los Angeles County (Shimizu *et al.*, 1987; Whittemore *et al.*, 1990). While there have been numerous reports on the more established Chinese and Japanese populations living in Los Angeles as well as elsewhere in the United States, there are no systematic data on cancer incidence patterns among the numerically less important Asian groups in Los Angeles County, especially those groups whose populations have increased only recently due to substantial in-migration. Vietnamese represent one such group. In fact, not only do there exist no data on Vietnamese immigrant populations, there are few data even on cancer patterns among Vietnamese in Vietnam. In light of the recent influx of a sizable Vietnamese population into Southern California, evaluation of their specific cancer patterns is now feasible.

We addressed this question by analysing data from the CSP. The CSP identifies all newly diagnosed cancer cases occurring among the now more than 8.8 million residents of Los Angeles County. Since June, 1987, the CSP has been one of the ten regional registries of the California Tumor Registry, a population-based registry for the State of California. Well over 95% of the incident cancer cases occurring in Los Angeles County residents since 1972 have been identified. A detailed description of the methodology, organisation and administration of the CSP has been published elsewhere (Mack, 1977). Our analysis covers 20 anatomical sites for cancers diagnosed among Vietnamese and Chinese inhabitants of Los Angeles County from 1972-1988. We have chosen to include Chinese as well as Vietnamese in this report, because the geographic proximity of the two countries makes Chinese a pertinent comparison group. During this period, cancer patients were identified from systematic searches of hospital and non-hospital pathology files, as well as from routine screening of death certificates. About 2% of cases are identified solely from the latter source. For each cancer patient, address, birthdate, race, ethnicity, sex, site and histology (using International Classification of Diseases for Oncology (ICDO) topographical and morphological codes), and other pertinent data are abstracted from medical records. The pathology report is routinely copied and attached to the completed cancer abstract.

We selected all cancer cases occurring in Los Angeles County residents who were coded as Vietnamese, based either on birthplace or a special CSP ethnicity code which the registry has designated 'best guess' ethnicity. This designation is assigned based on a review of race, birthplace, surname and first name as primary factors, and religion and address as secondary ones.

Correspondence: R.K. Ross, Norris Cancer Hospital, 1441 Eastlake Avenue Suite 803, Los Angeles, CA 90033, USA.
Received 1 November 1990; and in revised form 19 February 1991.

There were estimated to be 26,000 Vietnamese in Los Angeles County in 1980 and by 1986, Heer and Herman estimated this population to have grown to nearly 44,000. Because Vietnamese immigrants are a recent addition to the Los Angeles County populace, there were no adequate age- and year-specific denominator data at our disposal for the calculation of incidence rates. Therefore we analysed the data using Proportional Incidence Ratios (PIRs) for cancer occuring at each of the selected sites, within the Vietnamese and Chinese populations. PIR calculations enabled us to create ratios for comparison within each category of interest based on the cancer site distribution pattern among all racial-ethnic groups in Los Angeles County during the same period. The PIRs were calculated by dividing the total number of observed cancers in each particular site for all ages within each of the two race-ethnicity groups (Vietnamese and Chinese), by the total number of expected cases (with the same parameters) and multiplying by 100. The expected number of cancers for a particular race, site, and age group was derived from the product of the total number of cancers of all sites in that race and age group and the ratio of the total number of cancers of the particular site in that age group among all races to the total number of cancer of all sites in that age group among all races. The age-specific results were then summed over all age groups to obtain the final values for our comparisons.

Ninety-five per cent confidence limits for the race- and site-specific PIRs were calculated assuming that the observed number of cases (numerator) follows a Poisson distribution, then taking advantage of the exact relationship between the Poisson and the Chi-square distribution (Mulder, 1983).

The total number of cases for each cancer site and the associated PIRs are shown in Table I. Both Vietnamese and Chinese men and women demonstrate exceedingly high PIRs

**Table I** Proportional Incidence Ratios (PIR) and 95% Confidence Intervals (CI) for cancer among Vietnamese and Chinese men in Los Angeles County, 1972-1988. (*n* = total cases)

| | Vietnamese | | Chinese | |
|---|---|---|---|---|
| *Site* | *n* | *PIR (95% CI)* | *n* | *PIR (95% CI)* |
| Nasopharynx | 9 | 1039 (475,1972) | 71 | 1705 (1332,2151) |
| Oesophagus | 9 | 311 (142,590) | 21 | 111 (69,170) |
| Stomach | 22 | 281 (176,425) | 85 | 168 (134,207) |
| Colon | 16 | 77 (44,125) | 192 | 139 (120,160) |
| Rectum | 15 | 137 (77,227) | 111 | 159 (131,191) |
| Liver | 27 | 1049 (691,1526) | 108 | 719 (588,866) |
| Larynx | 5 | 98 (32,229) | 16 | 50 (29,81) |
| Lung | 51 | 121 (90,158) | 282 | 102 (91,115) |
| Prostate | 7 | 21 (9,44) | 141 | 58 (48,68) |
| Bladder | 77 | 47 (19,98) | 73 | 74 (58,93) |
| Kidney | 3 | 46 (10,134) | 28 | 72 (48,104) |
| Nervous system | 3 | 46 (10,134) | 22 | 74 (47,113) |
| Thyroid | 2 | 64 (8,231) | 18 | 142 (84,224) |
| Hodgkin's | 2 | 43 (5,156) | 2 | 12 (2,44) |
| Non-Hodgkin's lymphoma | 11 | 113 (56,202) | 41 | 81 (58,110) |
| Multiple myeloma | 3 | 121 (25,352) | 15 | 92 (51,152) |
| Leukaemia | 10 | 102 (49,187) | 39 | 79 (57,109) |

for nasopharyngeal cancer and liver cancer. Vietnamese men and women both show substantially elevated PIRs for oesophageal and stomach cancer; in both instances these were substantially higher than the associated PIRs in Chinese. Vietnamese and Chinese show approximately the expected rates of lung cancer but both show quite low rates of bladder cancer, another smoking-related site. PIRs for most hormone-related cancers – prostate, breast and corpus – are low in both groups but especially among Vietnamese. An exception for both of these racial-ethnic groups is ovarian cancer. The most prevalent cancer among Vietnamese women is cervix cancer, and the associated PIR is substantially elevated.

Our results show a number of similarities between Vietnamese cancer patterns and those among Chinese in Los Angeles, as well as those among native Southern Chinese. One explanation for this phenomenon is that Cantonese of South China have migrated to Vietnam, and that the cancer patterns we are observing are a reflection of those among transplanted Chinese, rather than those indigenous to Vietnamese. To guard against this possibility we carefully re-reviewed each surname among Vietnamese cancer patients in our registry. Only a few had surnames which were clearly or possibly Chinese.

The high rates for several of the cancers common to both groups can be attributed to common environmental exposures. Thus, the high incidence of liver cancer in both populations is likely readily explained by a high prevalence of chronic infections with hepatitis B virus throughout South-east Asia (Yeh *et al.*, 1989). We expect that the nearly equal relatively high lung cancer rates reflect high rates of smoking common to many Asian populations (Yu & Henderson, 1990). It is noteworthy, however, that bladder cancer, which has often been linked to smoking, is virtually non-existent in immigrant Vietnamese females and quite rare among immigrant Vietnamese males. A similar phenomenon (high lung cancer rates, low bladder cancer rates) has been observed in other Asian populations, such as Chinese men in Shanghai (WHO, 1982). Low rates of hormone-related cancers (i.e. those for breast, prostate, and corpus uteri) are also common to many Asian populations (WHO, 1982).

However, we were surprised by the high rates of both nasopharyngeal and stomach cancer common to both migrant groups in Los Angeles. There is growing evidence that the principal cause of cancer of the nasopharynx in South China is consumption of Cantonese-style salted fish (Yu & Henderson, 1990), a dietary practice not common to Vietnamese. The high rates of stomach cancer among Chinese is also presumably dietary in origin, but the precise dietary factors remain unknown.

Parkin published data on the distribution of cancer cases from a hospital-based series in Ho Chi Minh City Hospital from 1976–1986 (Parkin, 1986). The most striking finding in that series was the very high prevalence of cervix cancer, which accounted for over 53% of all cancer cases in women. Parkin speculated that this series might overestimate the true importance of cervix cancer due to selection factors leading to admission into the Ho Chi Minh City Hospital, which has departments of both gynecology and radiotherapy (Parkin, 1986). Nonetheless, we confirm the important contribution of cervix cancer to the overall malignancy profile among female Vietnamese immigrants to Southern California. Although breast cancer in women, lung cancer in men and cancer of the nasopharynx in both sexes also made substantial contributions to the cancer burden in that series, liver cancer, esophageal cancer, and stomach cancer were all relatively uncommon, compared to their important contribution to overall cancer occurrence in Vietnamese immigrants. Prostate cancer and bladder cancer were both exceedingly rare in that hospital-based series as they are in Los Angeles.

**Table II** Proportional Incidence Ratios (PIR) and 95% Confidence Intervals (CI) for Cancer among Vietnamese and Chinese women in Los Angeles County, 1972–1988. (*n* = total cases)

| Site | Vietnamese *n* | Vietnamese *PIR (95% CI)* | Chinese *n* | Chinese *PIR (95% CI)* |
|---|---|---|---|---|
| Nasopharynx | 6 | 1292 (474,2812) | 38 | 2151 (1522,2952) |
| Oesophagus | 3 | 233 (48,682) | 7 | 93 (37,192) |
| Stomach | 26 | 575 (376,843) | 59 | 229 (175,296) |
| Colon | 16 | 80 (46,130) | 117 | 99 (82,119) |
| Rectum | 7 | 87 (35,178) | 68 | 146 (114,186) |
| Liver | 5 | 432 (140,1008) | 31 | 515 (350,732) |
| Larynx | 2 | 164 (20,593) | 0 | 0 (0,58) |
| Lung | 17 | 78 (46,125) | 123 | 102 (85,122) |
| Breast | 45 | 53 (38,70) | 362 | 90 (81,100) |
| Cervix | 46 | 255 (187,340) | 82 | 130 (103,161) |
| Corpus uteri | 8 | 38 (16,75) | 63 | 58 (44,74) |
| Ovary | 20 | 139 (85,214) | 77 | 119 (93,148) |
| Bladder | 0 | 0 (0,79) | 24 | 86 (55,128) |
| Kidney | 4 | 126 (34,323) | 15 | 85 (48,141) |
| Thyroid | 15 | 153 (86,253) | 35 | 110 (77,154) |
| Hodgkin's | 0 | 0 (0,109) | 6 | 57 (21,123) |
| Non-Hodgkin's lymphoma | 9 | 127 (58,241) | 47 | 131 (96,174) |
| Multiple myeloma | 4 | 208 (57,533) | 9 | 79 (36,150) |
| Leukaemia | 8 | 136 (59,267) | 27 | 89 (59,130) |
| Nervous System | 3 | 67 (14,197) | 15 | 77 (43,128) |

This work was supported by grant CA17054 from the National Institutes of Health and by Subcontract 050E-8709 with the California Public Health Foundation, which is supported by the California Department of Health Services as part of its statewide cancer reporting program mandated by Health and Safety Code Section 210 and 211.3. The ideas and opinions expressed herein are those of the author, and no endorsement by the State of California, Department of Health Services or the California Public Health Foundation is intended or should be inferred.

### References

HEER, D.M. & HERMAN, P. (1990). *A Human Mosaic: An Atlas of Ethnicity in Los Angeles County, 1980–1986*. Western Economic Research Company Inc.

MACK, T.M. (1977). Cancer Surveillance Program in Los Angeles County. *Natl Cancer Inst. Monogr.*, **47**, 99.

MULDER, P.G.H. (1983). An exact method for calculating a confidence interval of a Poisson parameter. *Am. J. Epidemiol.*, **117**, 377.

PARKIN, D.M. (1986). Vietnam, Ho Chi Minh City 1976–1981 (Luong Tan Truong). In *Cancer Occurrences in Developing Countries*, Parkin, D.M. (ed.), pp. 309.

SHIMIZU, H., MACK, T.M., ROSS, R.K. & HENDERSON, B.E. (1987). Cancer of the gastrointestinal tract among Japanese and white immigrants in Los Angeles County. *J. Natl Cancer Inst.*, **78**, 223.

WHITTEMORE, A.S., WU, A.H., SHU, Z. & 12 others (1990). Diet, physical activity and colorectal cancer among Chinese in North America and the People's Republic of China. *J. Natl Cancer Inst.*, **82**, 915.

XU, Z.Y., BLOT, W.J., XIAO, H.P. & 7 others (1989). Smoking, air pollution and the high rates of lung cancer in Shenyang, China. *J. Natl Cancer Inst.*, **81**, 1806.

YEH, F.S., YU, M.C, MO, C.C., LUO, S., TONG, M.J. & HENDERSON, B.E. (1989). Hepatitis B virus, aflatoxins, and hepatocellular carcinomas in Guangxi, China. *Cancer Res.*, **49**, 2506.

YU, M.C. & HENDERSON, B.E. (1990). Nasopharynx. In *Cancer Epidemiology and Prevention*. Schottenfeld, D. & Fraumeni, J.F. (eds). W.B. Saunders Company: Philadelphia, (in press).

WORLD HEALTH ORGANIZATION (1983). *Cancer Incidence in Five Continents*. Vol. V. Muir, C., Waterhouse, J., Mack, T., Powell, J. & Whelan, S. (eds). International Agency for Research on Cancer. Publication No. 88: Lyon.